# Efficacy and safety of the flexible negative-pressure ureteral sheath in retrograde intrarenal surgery: a systematic review and meta-analysis

**DOI:** 10.3389/fsurg.2025.1649574

**Published:** 2025-08-05

**Authors:** Lin Wang, Zhifang Luo, Wen Huang, Qilei Jia

**Affiliations:** ^1^ Department of Basic Medicine, Chengdu University, Chengdu, Sichuan, China; ^2^ Department of Urology, People’s Hospital of Qingbaijiang District, Chengdu, Sichuan, China

**Keywords:** flexible and navigable suction, retrograde intrarenal surgery, urinary calculi, renal calculi, meta-analysis.

## Abstract

**Purpose:**

To evaluate the safety and efficacy of the flexible negative-pressure ureteral sheath (FANS) in flexible ureteroscopic lithotripsy (RIRS) for urinary calculi.

**Methods:**

Computerized searches were performed in English databases including PubMed, Embase, Web of Science, and the Cochrane Library up to 4 February 2025, to identify clinical studies on the FANS combined with RIRS for urinary calculi. Data analysis and extraction were conducted using Stata 18.0 and Review Manager 5.3 software.

**Results:**

This meta-analysis of nine studies (1,785 patients) showed that the FANS significantly improved stone-free rates [odds ratio (OR) = 2.58, 95% CI = 2.11–3.15] and reduced intraoperative complications (OR = 0.32, *P* = 0.02), postoperative complications (OR = 0.37), reoperation (OR = 0.28), and stone basket use (OR = 0.01) when compared with the traditional ureteral access sheath (T-UAS). Subgroup analyses confirmed the superiority of the FANS in removing stones ≤20 mm (OR = 2.10) and >20 mm (OR = 3.03), with shorter operative times for small stones (SMD = −0.31) and Ho:YAG (SMD = −0.63).

**Conclusions:**

The FANS enhances RIRS efficacy and safety by improving stone clearance, reducing complications, and minimizing auxiliary instrument use. While it did not shorten hospitalization or overall operative time, its advantages in removing larger stones and laser compatibility underscore its clinical value.

**Systematic review registration:**

https://www.crd.york.ac.uk/prospero/, identifier (CRD42024611779).

## Introduction

Urolithiasis is a prevalent chronic condition worldwide, with a reported rate of incidence of up to 15%. Retrograde intrarenal surgery (RIRS) has gained widespread clinical acceptance due to its minimally invasive nature, faster recovery, and lower complication rates. Compared with percutaneous nephrolithotomy (PCNL), RIRS results in significantly less surgical trauma and a shorter postoperative hospital stay (1). Moreover, it offers superior stone clearance and reduced recurrence rates relative to shock wave lithotripsy (SWL) (2). Accordingly, the European Association of Urology (EAU) guidelines recommend RIRS as the first-line treatment for upper urinary tract stones measuring less than 2 cm in diameter (3, 4).

Among the technological innovations that have advanced RIRS, the flexible negative-pressure ureteral access sheath (FANS) has emerged as a particularly promising adjunct. The FANS combines a pliable access sheath with integrated negative-pressure suction functionality (5). Key structural innovations include a steerable distal tip for improved access to complex calyceal anatomy, a dual-lumen configuration enabling simultaneous irrigation and aspiration, and an atraumatic tip design to minimize mucosal injury (6). These features are designed to enhance maneuverability, maintain low and stable intrarenal pressure (IRP), and facilitate real-time evacuation of stone fragments (7). The resultant improvements in surgical visibility, intrarenal pressure control, and debris clearance are hypothesized to reduce postoperative infection rates and other procedure-related complications (8, 9). Clinically, the FANS has shown potential in improving stone-free rates (SFRs), shortening operative time, and reducing reliance on auxiliary interventions. However, published outcomes remain inconsistent with respect to efficacy and safety indicators such as the SFR, intraoperative blood loss, operative duration, and complication rates (10–12). Therefore, this systematic review and meta-analysis aims to synthesize current evidence to comprehensively evaluate the clinical efficacy and safety of the FANS in RIRS, with the goal of informing evidence-based clinical decision-making.

## Methods

### Search strategy

A systematic literature search was performed across four major databases (PubMed, Embase, Web of Science, and MEDLINE) from their inception to 4 February 2024, utilizing a combination of Medical Subject Headings (MeSH) terms and free-text keywords. The search strategy included three conceptual domains: (1) interventions: “Ureteroscopy” OR “flexible ureteroscopy” OR “retrograde intrarenal surgery”; (2) diseases: “Calculi” OR “kidney stone” OR “ureteral stone”; (3) technological Features: “Pliability” OR “Flexibility” OR “Vacuum” OR “Suction”. Boolean operators (AND/OR) were systematically applied to refine the search syntax. This meta-analysis adhered to PRISMA 2020 guidelines and was prospectively registered on PROSPERO (CRD42024611779).

### Inclusion and exclusion criteria

Inclusion criteria: Studies were eligible if they met the following criteria: (1) study design: Prospective comparative studies or non-randomized comparative designs, including prospective/retrospective cohort studies, case-control studies, or historical controlled trials; (2) population: Adults (≥18 years) diagnosed with upper urinary tract calculi (renal or ureteral stones) undergoing RIRS; (3) intervention: The experimental group utilized the FANS, defined as a ureteral access sheath with tip-adjustable flexibility and integrated negative-pressure suction. The control group employed the traditional UAS (T-UAS), with or without suction capability. To minimize confounding, only studies in which both groups used the same laser type (either TFL or Ho:YAG) were included. Variations in auxiliary tools (e.g., stone baskets) were allowed and addressed through subgroup analyses; (4) outcomes: Primary outcome: the SFR assessed by imaging (CT/KUB) with defined residual fragment thresholds (≤3 or ≤2 mm); secondary outcomes: complications, stone basket utilization, reoperation rates, operative time, hospitalization duration, hemoglobin decline rate, and intraoperative adverse events.

Exclusion criteria: (1) correspondences, review papers, laboratory investigations, case reports, and animal experimental studies. (2) Absence of crucial information such as sample size, 95% confidence interval, and *P*-value, or incapability of reasonably converting and computing these values. (3) Inability to access the original data, duplicate literature. (4) Non-English language publications were excluded.

### Quality assessment

Two independent investigators conducted the quality assessment using standardized Cochrane Risk of Bias (RoB) tools, with cross-validation to ensure consistency. Discrepancies were resolved through consultation with a third reviewer. Data extractors were blinded to authors and journal affiliations to minimize bias. Methodological rigor was evaluated using the following: (1) ROBINS-I (Risk Of Bias In Non-randomized Studies of Interventions) for non-randomized studies (NRSIs), assessing seven domains: confounding, selection bias, intervention classification, deviations from intended interventions, missing data, outcome measurement, and selective reporting. (2) RoB 2.0 for randomized controlled trials (RCTs), evaluating randomization processes, deviations from interventions, missing outcome data, outcome measurement, and selective reporting (13, 14).

### Data extraction

Data extraction encompassed study characteristics (first author, study design), intervention details (experimental/control groups, sample size, laser type), baseline parameters (stone size, patient age), stone-free criteria (imaging modality, residual fragment thresholds, and timing of evaluation), and outcome measures (stone-free rate, complications, stone basket utilization, reoperation rate, operative time, hospitalization duration, hemoglobin decline).

The specific time points at which CT or KUB was performed to assess stone-free status were extracted for each study. These assessments occurred at varying postoperative intervals—typically at ≤24 h (Early Stage), 1–2 months (Middle Stage), or 3 months (Long-term Stage)—and are detailed in Table 1. We adopted the original definitions and timing reported by the included studies without imposing a uniform evaluation schedule. For analytical consistency, these time points were grouped into three predefined intervals corresponding to early stage, middle stage, and long-term stage follow-up periods during subgroup analysis. Potential heterogeneity due to these differences was considered during data synthesis.

### Statistical and meta-analysis

Data analysis was performed using Stata 18.0 and Review Manager Version 5.3 software. Dichotomous outcomes (stone-free rate, complications) were expressed as odds ratios (ORs) with 95% confidence intervals (CIs), while continuous variables (operative time, hemoglobin decline) were analyzed using standardized mean differences (SMDs). Pooled effect estimates were evaluated with *Z*-tests, and statistical significance was defined as a two-tailed *P* < 0.05. Heterogeneity was assessed via the *I*^2^ statistic, with *I*^2^ > 50% indicating substantial heterogeneity and warranting a random-effects model; otherwise, a fixed-effects model was applied. Sensitivity analyses were performed to examine the stability of pooled results by sequentially excluding individual studies (15). Subgroup analyses were stratified by stone clearance time (postoperative ≤24 h, 1–2, and 3 months), stone size (≤20, >20 mm), and laser type (TFL, Ho:YAG). Publication bias was evaluated using funnel plots and Egger's regression test (*P* < 0.05 considered significant) (16, 17).

## Results

### Characteristics of eligible studies

**Figure 1:**
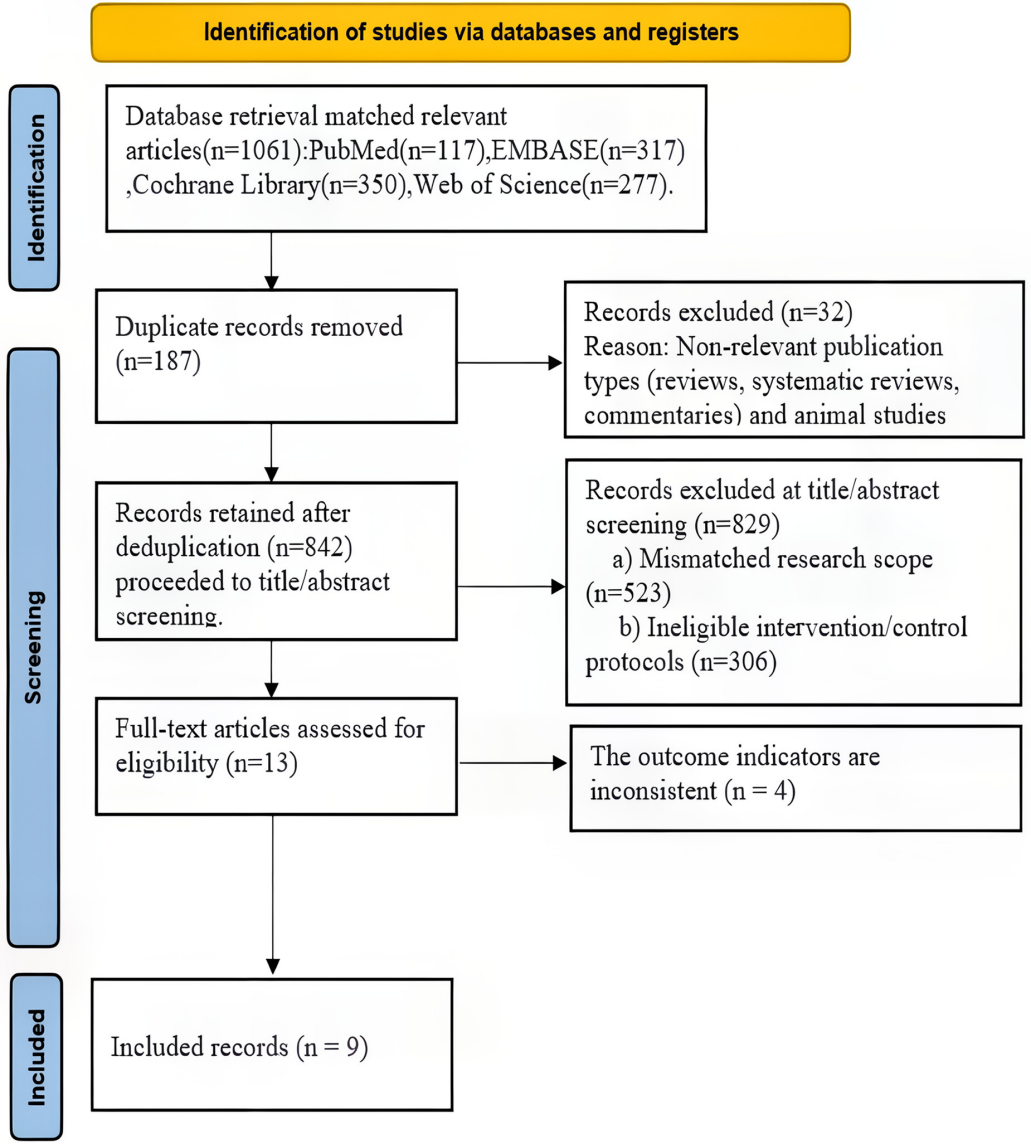
Literature screening flow chart.

After an initial search that identified 1,061 related studies, and after excluding ineligible literature according to standards, a total of nine eligible studies (18–26) were ultimately included (Figure 1).

### Quality of eligible studies

**Figure 2:**
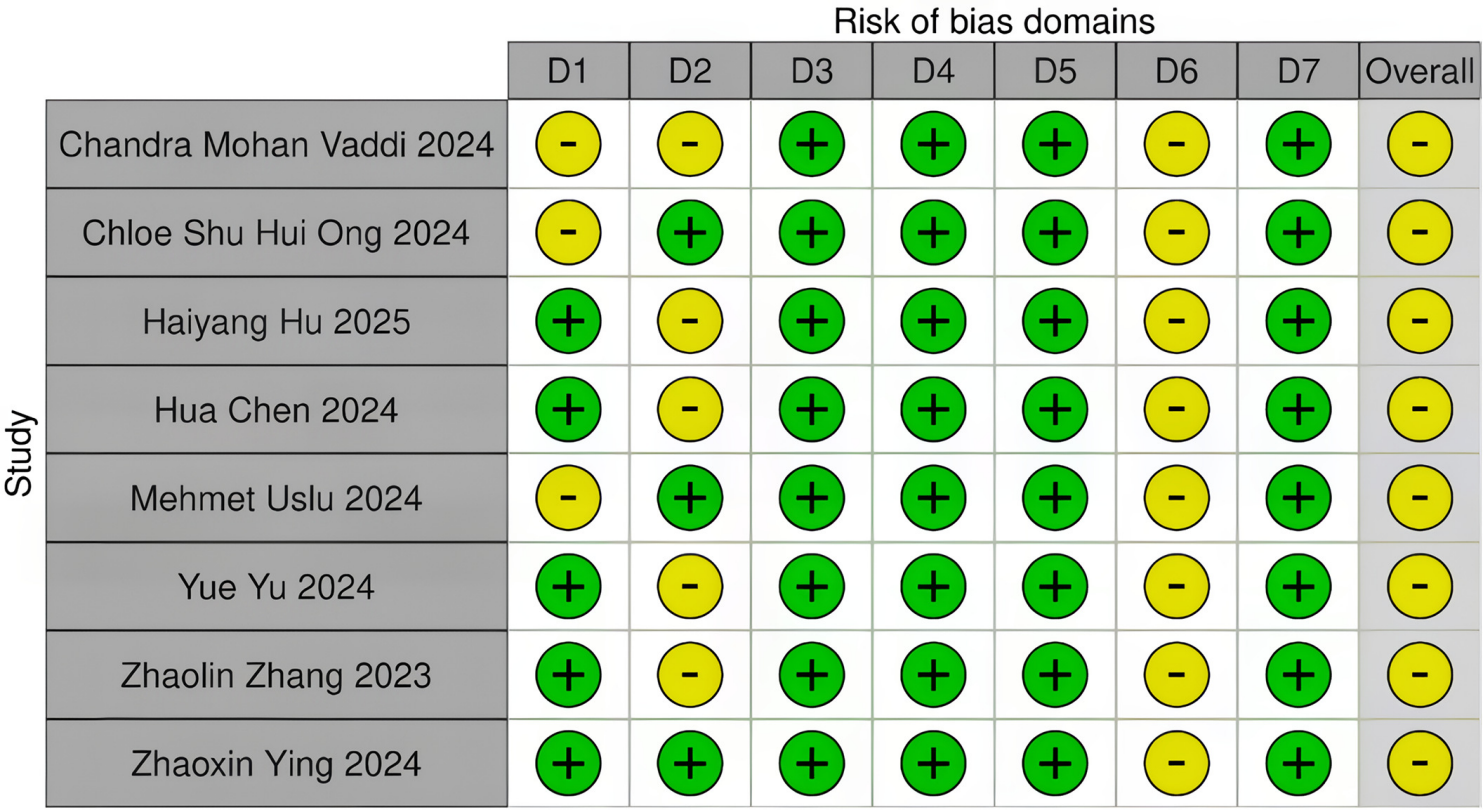
Summary of each retrospective study evaluated using ROBINS-I.

**Figure 3:**
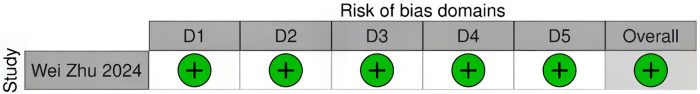
Risk of the bias graph of each RCT appraised by RoB 2.

A total of nine literature studies (18–26) were included, involving 1,785 patients and 1,785 operations. Among them, 851 patients used the FANS, and 934 patients adopted traditional ureteroscope sheaths. The included studies comprised one prospective randomized controlled study (26), seven non-randomized controlled studies (18, 19, 21–25), and one historical controlled study (20) (Table 1). The ROBINS-I and RoB 2 tools from the Cochrane Collaboration Network (13, 14) were used for bias risk assessment: For eight non-randomized interventional studies, ROBINS-I analyzed seven domains (D1–D7), showing all studies had an overall moderate bias risk. Specific manifestations were as follows: some studies had a moderate risk in D1 (Chandra Mohan Vaddi, Chloe Shu Hui Ong, Mehmet Uslu); most studies presented a moderate risk in D2 (Haiyang Hu, Yue Yu); all studies had a moderate risk in D6. For one prospective randomized controlled study, RoB 2.0 analyzed five domains (D1–D5), indicating a low overall bias risk with high-quality study design and implementation (Tables 2, 3, Figures 2, 3).

**Table 1:** Basic characteristics of included studies.

Author/year	Study design	Group (T/C)	Sample (M/F)	Laser type	Stone size (mm)*	Age*	Stone-free criteria (imaging modality and time)	Outcomes
Vaddi et al. (20)	Historical controlled trial	Flexible Ureteral Access Sheath with Suction	33/17	TFL	14.2 (5.3)	42.2 (14.6)	Fragments <2 mm (NCCT KUB) at 2 months	①②④⑤⑧
		T-UAS	38/12		14.8 (3.5)	45.4 (11.8)		
Ong et al. (21)	Multicenter retrospective	Flexible and Navigable Suction Ureteral Access Sheath	25/20	TFL	16.0 (1.5)	53.5 (6.7)	100% SFR (NCCT) within 6 weeks	①②③④⑤⑧
		T-UAS	25/20		13.5 (1.2)	50.0 (5.5)		
Hu et al. (18)	Retrospective cohort	Tip-flexible Suctioning Ureteral Access Sheath	46/32	Ho:YAG	16.5 (1.2)	49.5 (5.7)	No fragments or <2 mm (US/CT) at immediate post-op and 1 month	①②④⑤
		T-UAS	70/57		15.5 (1.2)	53.5 (3.2)		
Chen et al. (22)	Case control	Tip-flexible Suctioning Ureteral Access Sheath	76/49	Ho:YAG	28.1 (1.5)	45.6 (12.9)	<2 mm fragments (KUB/CT) at POD1 and CT at 1 month	①②④⑤⑥⑧
		T-UAS	65/48		25.2 (3.7)	46.3 (14.8)		
Uslu et al. (19)	Prospective data analysis	Novel Tip-bendable Suction Ureteral Access Sheath	29/14	Ho:YAG	12.5 (2.2)	54.0 (7.5)	≤3 mm fragments (NCCT) at 1 month	①②⑤⑥
		T-UAS	27/18		10.5 (1.2)	51.5 (4.7)		
Zhu et al. (26)	RCT	Tip-bendable Suction Ureteral Access Sheath	89/71	Ho:YAG	14.0 (7.4)	53.0 (14.0)	Endoscopic clearance <2 mm (KUB/US) at 24 h; No >2 mm fragments (low-dose CT) at 3 months	①②③④⑤⑥⑦
		T-UAS	96/64		11.0 (5.1)	52.0 (16.1)		
Yu et al. (23)	Case control	Novel Flexible Ureteral Access Sheath	75/77	Ho:YAG	15.5 (2.0)	51.1 (12.2)	No fragments (NCCT) at POD1 and 1 month; Additional US/KUB for outpatient follow-up	①②③⑤⑥⑦
		T-UAS	80/72		15.2 (1.9)	50.5 (11.8)		
Zhang et al. (24)	Case control	Novel Tip-flexible Suctioning Ureteral Access Sheath	55/47	Ho:YAG	18.4 (4.6)	47.6 (9.1)	<2 mm fragments (KUB/CT) with CT confirmation for suspected cases	①②④⑤⑥⑦
		T-UAS	71/41		18.2 (4.4)	46.7 (11.8)		
Ying et al. (25)	Retrospective cohort	Tip-flexible Suctioning Ureteral Access Sheath	65/38	Ho:YAG	15.5 (5.8)	53.0 (12.9)	No fragments or <2 mm (KUB/NCCT) at POD1 and 3 months	①②③⑤⑥
		T-UAS	103/35		15.6(6.4)	53.9(14.7)		

* Data presented as Mean (SD), ① Stone-free rate, ② Postoperative Complications, ③ Stone basket usage, ④ Reoperation, ⑤ Operation time, ⑥ Hospital Stay, ⑦ Hemoglobin loss rate, ⑧ Intraoperative complications.

T, experimental group; C, control group; TFL, thulium fiber laser; Ho:YAG, Holmium:Yttrium Aluminum Garnet; T-UAS, traditional ureteral access sheath; CT, computed tomography; KUB, Kidneys, Ureters, and Bladder (plain film); NCCT, noncontrast computed tomography; RCT, randomized controlled trial; POD, postoperative day; US, ultrasonography.

**Table 2:** Quality appraisal of non-randomized studies (ROBINS-I domains).

Study	D1	D2	D3	D4	D5	D6	D7	Overall
Vaddi et al. (20)	Moderate	Moderate	Low	Low	Low	Moderate	Low	Moderate
Ong et al. (21)	Moderate	Low	Low	Low	Low	Low	Low	Moderate
Hu et al. (18)	Low	Moderate	Low	Low	Low	Moderate	Low	Moderate
Chen et al. (22)	Low	Moderate	Low	Low	Low	Moderate	Low	Moderate
Uslu et al. (19)	Moderate	Low	Low	Low	Low	Moderate	Low	Moderate
Yu et al. (23)	Low	Moderate	Low	Low	Low	Moderate	Low	Moderate
Zhang et al. (24)	Low	Moderate	Low	Low	Low	Moderate	Low	Moderate
Ying et al. (25)	Low	Low	Low	Low	Low	Moderate	Low	Moderate

**Table 3:** Risk of bias assessment for randomized controlled trials (RoB 2.0 criteria).

Study	D1	D2	D3	D4	D5	Overall
Zhu et al. (26)	Low	Low	Low	Low	Low	Low

### Main results of meta-analysis

#### Stone-free rate

**Figure 4:**
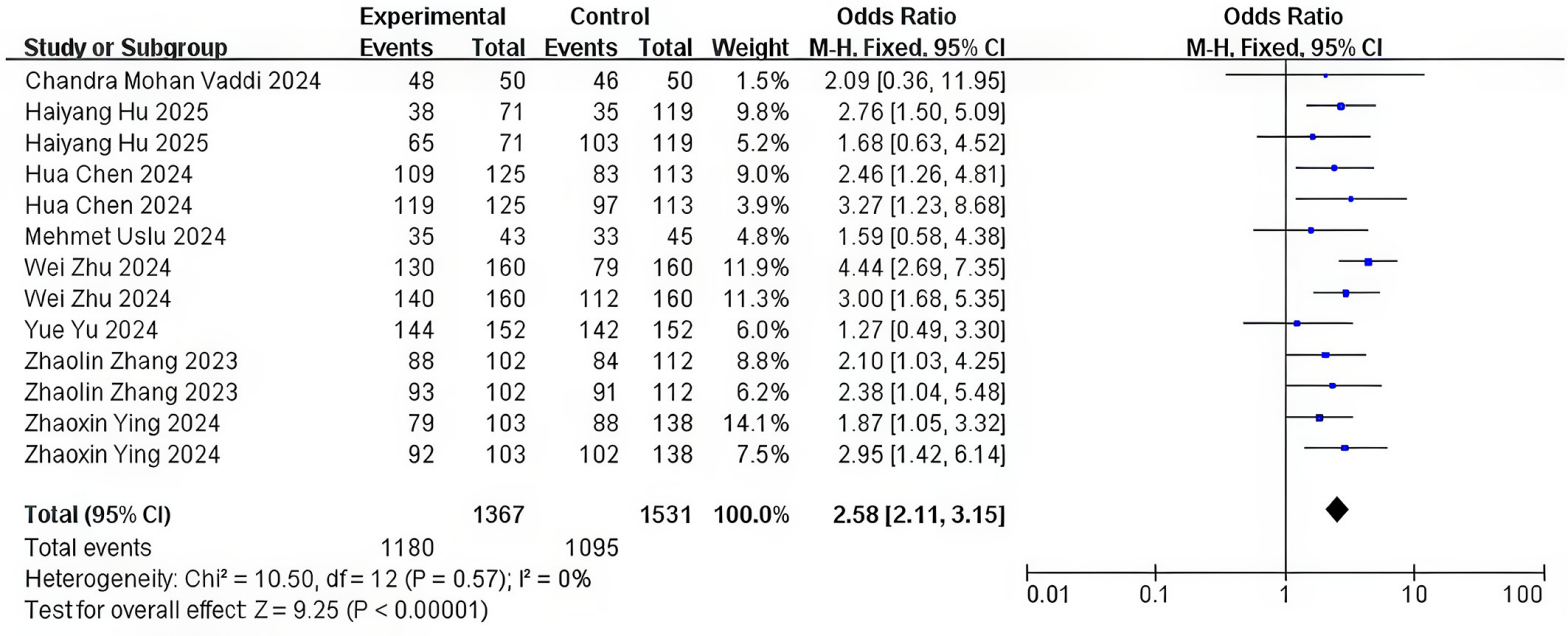
Stone-free rate.

The nine included studies exhibited variability in SFR definitions, primarily differing in follow-up timing (postoperative day 1–3 months) and imaging modalities (NCCT, KUB, US, or endoscopy) with residual fragment thresholds of <2 or ≤3 mm. Initial pooled analysis revealed substantial heterogeneity (*I*^2^ = 82%, *P* < 0.0001). Sensitivity analysis identified two outliers contributing to heterogeneity Chloe Shu Hui Ong and Yue Yu (21, 23). After excluding these studies, heterogeneity resolved (*I*^2^ = 0%, *P* = 0.57). Fixed-effects meta-analysis of the remaining seven studies demonstrated a superior SFR with flexible negative-pressure sheaths versus the T-UAS (OR = 2.58, 95% CI = 2.11–3.15, *P* < 0.0001) (Figure 4).

#### Intraoperative complications

Three studies reported intraoperative complications (ureteral injury, pelvic perforation, or bleeding) (20–22). Pooled analysis revealed low heterogeneity (*I*^2^ = 0%, *P* = 0.44), with the FANS group demonstrating significantly lower complication rates than the T-UAS group (OR = 0.32, 95% CI = 0.12–0.82, *P* = 0.020) (Table 4).

**Table 4:** Complications.

Outcome	Studies (*n*)	Heterogeneity		95% CI	*P*
		*P*	*I*^2^ (%)		
Intraoperative complications	3	0.440	0	0.32 (0.12–0.82)	0.020
Postoperative complications	9	0.070	44	0.37 (0.28–0.48)	<0.0001
Clavien–Dindo Grade I–II	5	0.370	6	0.36 (0.24–0.54)	<0.0001
Clavien–Dindo Grade ≥III	5	0.820	0	0.28 (0.10–0.79)	0.020

#### Postoperative complications

Pooled analysis of nine studies demonstrated moderate heterogeneity (*I*^2^ = 44%, *P* < 0.0001) in postoperative complication reporting. Four studies utilized the Clavien–Dindo classification (20, 21, 24, 26), two employed modified versions (18, 19), and three reported complications descriptively (22, 23, 25). Enabling fixed-effects analysis: the FANS significantly reduced overall complications when compared with the T-UAS (OR = 0.37, 95% CI = 0.28–0.49, *P* < 0.0001). Subgroup analysis by Clavien–Dindo severity revealed the following: Grades 1–2: Lower incidence with the FANS (OR = 0.36, 95% CI = 0.24–0.54, *P* < 0.0001; *I*^2^ = 6%). Grades ≥3: Lower incidence with the FANS (OR = 0.28, 95% CI = 0.10–0.79, *P* = 0.020; *I*^2^ = 0%) (Table 4).

### Surgical-related indicators

#### Hospital stay

Six studies reported hospitalization duration (18, 19, 23–26), with one study (26) defining it as the interval from surgery to discharge. Pooled analysis showed no heterogeneity (*I*^2^ = 0%, *P* = 0.840) and no significant difference between the FANS and the T-UAS groups (SMD = −0.08, 95% CI = −0.18–0.03, *P* = 0.150; fixed-effects model) (Table 5).

**Table 5:** Surgical outcomes.

Outcome	Studies (*n*)	Heterogeneity		95% CI	*P*
		*P*	*I*^2^ (%)		
Hospital stay	6	0.84	0	−0.08 (−0.18–0.03)	0.150
Operative time	9	<0.0001	98	−0.30 (−0.94–0.34)	0.360
Hemoglobin loss	3	0.01	77	0.16 (−0.12–0.44)	0.270
Reoperation	6	0.19	33	0.28 (0.15–0.54)	0.0001
Stone basket usage	4	0.0006	83	0.01 (0.00–0.08)	<0.0001

#### Operation time

Nine studies were included, with varying definitions (e.g., stone extraction time, scope insertion to stent placement). High heterogeneity (*I*^2^ = 98%, *P* < 0.0001) necessitated a random-effects model. No intergroup difference was observed (SMD = −0.30, 95% CI = −0.94–0.34, *P* = 0.360) (Table 5).

#### Hemoglobin loss rate

Three studies (23, 24, 26) reported hemoglobin decline, with significant heterogeneity (*I*^2^ = 77%, *P* = 0.010). A random-effects analysis revealed no significant difference (SMD = 0.16, 95% CI = −0.12–0.44, *P* = 0.270) (Table 5).

#### Reoperation rate

Six studies (18, 20, 21, 22, 24, 26) reported reoperations (causes: steinstrasse, residual fragments, subcapsular hematoma/stent migration). A fixed-effect analysis demonstrated lower reoperation rates with the FANS (OR = 0.28, 95% CI = 0.15–0.54, *P* = 0.0001; *I*^2^ = 33%) (Table 5).

#### Stone basket usage

Four studies (21, 23, 25, 26) showed high heterogeneity (*I*^2^ = 83%, *P* = 0.0006). The FANS significantly reduced basket utilization (OR = 0.01, 95% CI = 0.00–0.08, *P* < 0.0001; random-effects model) (Table 5).

#### Subgroup analyses

Subgroup analysis demonstrated that for stones ≤20 mm, the FANS group exhibited a significantly superior stone clearance rate (OR = 2.10, 95% CI = 1.55–2.84) and reduced postoperative complications (OR = 0.41, 95% CI = 0.30–0.57), operation time (SMD = −0.31, 95% CI = −0.45–−0.18), and reoperation rate (OR = 0.11, 95% CI = 0.03–0.39) compared with the T-UAS group (*P* < 0.050). For stones >20 mm, although the operation time in the FANS group was prolonged (SMD = 0.19, 95% CI = 0.05–0.33), advantages in stone clearance rate (OR = 3.03, 95% CI = 2.31–3.97) and complication control (OR = 0.29, 95% CI = 0.18–0.47) were still observed. Stratified by laser type, under the Ho:YAG laser, the FANS group presented lower postoperative complications (OR = 0.37, 95% CI = 0.27–0.50), operation time (SMD = −0.63, 95% CI = −1.35 to −0.08), and reoperation rate (OR = 0.46, 95% CI = 0.21–0.99). Under the TFL laser, the FANS group showed a significant reduction in the reoperation rate (OR = 0.09, 95% CI = 0.02–0.35). In the assessment of stone clearance time, the FANS group demonstrated significantly higher stone clearance rates than the control group in the early postoperative stage (≤24 h: OR = 2.74, 95% CI = 2.10–3.58), medium-term stage (1–2 months: OR = 1.98, 95% CI = 1.32–2.98), and long-term stage (≥3 months: OR = 2.98, 95% CI = 1.89–4.69) (*P* < 0.050). In addition, no statistical differences in hospital stay were observed across all subgroups (*P* > 0.050) (Table 6).

**Table 6:** Subgroup analyses.

Subgroup	Category	SFR	Postoperative complications	Operative time	Hospital stay	Reoperation
Stone size	≤20 mm	2.10 (1.55–2.84)*	0.41 (0.30–0.57)*	−0.31 (−0.45– −0.18)*	−0.10 (−0.26–0.05)	0.11 (0.03–0.39)*
	>20 mm	3.03 (2.31–3.97)*	0.29 (0.18–0.47)*	0.19 (0.05–0.33)*	−0.05 (−0.19–0.09)	0.47 (0.21–1.04)
Laser type	TFL	—	0.71 (0.37–1.38)	0.90 (−0.04–1.85)	—	0.09 (0.02–0.35)*
	Ho:YAG	2.65 (2.13–3.29)*	0.37 (0.27–0.50)*	−0.63 (−1.35–0.08)	−0.08 (−0.18–0.03)	0.46 (0.21–0.99)*
Clearance time	Early stage (≤24 h)	2.74 (2.10–3.58)*	—	—	—	—
	Middle stage (1–2 months)	1.98 (1.32–2.98)*	—	—	—	—
	Long-term stage (3 months)	2.98 (1.89–4.69)*	—	—	—	—

* *P* < 0.05.

## Discussion

Retrograde intrarenal surgery (RIRS), a form of natural orifice transluminal endoscopic surgery, has become an important approach for the treatment of upper urinary tract stones. In 2016, Professor Zeng Guohua from China reported a negative pressure flexible ureteroscope sheath. An oblique vent with pressure regulation function was added to the T-UAS (27), which was connected to a continuous negative pressure aspirator. This enabled the discharge of fragmented stones and irrigating fluid after laser lithotripsy from the gap between the sheath and the endoscope. This design has improved the stone clearance rate to a certain extent, enhanced the visual field, and may have reduced the intrarenal pelvic pressure (28). In 2023, Gauhar et al. reported about the FANS (29). With the popularization and application of the FANS, numerous clinical studies have investigated and observed its application effects. This study further analyzes the research results of nine literature studies.

### Stone-free rate

The present meta-analysis revealed that the SFR in the FANS group was significantly higher than that in the T-UAS group, and this finding was consistent with the results of studies by Liang et al. (30) The FANS effectively clears crushed stone particles through its negative-pressure suction function, reducing the accumulation of residual fragments. Notably, the definition of residual fragments has evolved from ≤4 mm to a stricter criterion of ≤2 mm (31). This improvement is attributed to the flexible tip design of the FANS, which allows active navigation into renal calyces and creates vortices that facilitate fragment aspiration, thus improving the single-stage SFR (32).

### Intraoperative complications

The intraoperative complication rate in the FANS group was significantly lower than that in the T-UAS group. This can be attributed to three key factors: (1) the 10-cm flexible tip of the FANS can actively/passively bend to better conform to the anatomical structures of the ureter and renal calyces, minimizing mechanical friction on the mucosa; (2) the negative-pressure function continuously clears laser-generated dust and fragments in real time, maintaining a clear operative field and reducing blind manipulation and tissue injury caused by visual obscuration; (3) the continuous suction creates a stable hydrodynamic environment that prevents retrograde migration of stone fragments into the renal pelvis or distal segments, thereby avoiding repetitive stone extraction maneuvers (32, 33). In addition, the negative-pressure suction maintains a low-pressure state in the renal pelvis, reducing the risk of renal pelvis perforation. These findings are consistent with previous biomechanical studies demonstrating improved mucosal protection through dynamic fluid management and sheath-tissue interaction optimization (34).

### Postoperative complications

Notwithstanding the Clavien–Dindo classification system, postoperative complications in the FANS group remained significantly lower than those in the T-UAS group, further confirming its safety profile. However, the occurrence of postoperative complications may be influenced by other surgery-related factors such as surgeon experience, perioperative care, and patient-specific characteristics. Therefore, future studies should incorporate these variables to conduct a more comprehensive evaluation of the clinical efficacy of the FANS.

### Surgical-related indicators

The rates of reoperation and stone basket utilization in the FANS group were lower than those in the T-UAS group. This advantage can be attributed to its integrated design of “stone fragmentation—dust suction—stone clearance,” which reduces the reliance on the passive stone retrieval mode (33–35). However, there were no significant differences between the two groups in terms of length of hospital stay, operation time, and the rate of hemoglobin decline. These non-significant findings may be associated with study heterogeneity, the degree of standardization of operations, and sample size.

### Subgroup analysis

#### Stone size subgroup

In both stone diameter ≤20 and >20 mm subgroups, the FANS group demonstrated significant advantages in the stone clearance rate and postoperative complications. This finding suggests that with the adoption of the FANS, the surgical indications for RIRS are expanding, and renal calculi >2 cm in diameter may now be managed through the FANS with reduced postoperative complications. This shift implies that select patients previously requiring percutaneous nephrolithotomy (PCNL) could transition to RIRS, aligning with the evolving trend toward natural orifice transluminal endoscopic surgery (29). Notably, the discrepancy in operation time correlated with stone volume: for ≤20 mm stones, the FANS significantly improved efficiency (SMD = −0.31) by reducing stone basket utilization and repetitive exploration. Conversely, for >20 mm stones, the time required for lithotripsy—especially for high-density or staghorn calculi—offset the efficiency gains from suction (SMD = 0.19) (30, 32). These results underscore the dual benefits of the FANS in terms of safety enhancement and indication expansion, while highlighting the need for tailored intraoperative strategies based on stone characteristics.

#### Laser type subgroup

The synergistic effect between the Ho:YAG laser and the FANS significantly improved the SFR (OR = 2.65). The pulsed energy delivery of Ho:YAG generates larger stone fragments, necessitating real-time suction by the FANS to prevent visual obstruction. Concurrently, the negative-pressure environment reduces thermal damage accumulation, further lowering complications (OR = 0.37). By contrast, the high ablation efficiency of the TFL laser (dust particle size <150 μm) theoretically makes it more compatible with the FANS, but this was not analyzed due to insufficient sample size. The discrepancy in postoperative complications (Ho:YAG: OR = 0.37; TFL: OR = 0.71) correlates with laser-tissue interaction mechanisms. The high peak power of Ho:YAG predisposes to local tissue edema, whereas the FANS mitigates intraoperative blind manipulation injuries by reducing intrapelvic pressure (IRP) and timely debris removal. TFL, with lower thermal damage and more uniform fragmentation, exerts minimal impact on intrarenal pressure, leading to non-significant complication differences. With regard to operation time, the non-significant reduction in the Ho:YAG group (SMD = −0.63) may be constrained by stone hardness and location, while in the TFL group, surgeon proficiency or laser stability may have masked the potential benefits of the FANS (SMD = 0.90) (33–36). These findings highlight the need for further investigation into laser-specific optimization strategies to maximize the clinical utility of the FANS. A recent multicenter prospective study further explored the performance of the FANS in comparison with the conventional UAS using both the Ho:YAG laser and the pulsed thulium laser (pTm:YAG) (39). Although promising in outcome trends, this study did not stratify results according to laser type per treatment group. As our meta-analysis strictly required uniform laser usage within study arms to reduce confounding bias, the data could not be incorporated into the pooled analysis. Nonetheless, this study underscores the growing interest in pTm:YAG as an alternative to traditional Ho:YAG, due to its high ablation precision and potential synergy with suction systems. Future research that clearly delineates group-specific laser usage is warranted to better elucidate the comparative effectiveness of different laser-FANS combinations.

#### Stone clearance time subgroup

The “continuous stone clearance” advantage of sustained postoperative negative-pressure suction of the FANS was evident across different time intervals. Early effects (≤24 h, OR = 2.74) were attributed to intraoperative immediate suction reducing residual fragment volume and preventing postoperative acute obstruction (e.g., steinstrasse formation). Medium-term benefits (1–2 months, OR = 1.98) were associated with negative-pressure promotion of urokinetic flushing, dislodging microresidual stones adhering to the mucosa. Long-term SFR improvement (≥3 months, OR = 2.98) resulted from the persistent action of the FANS on occult calyceal fragments. Notably, traditional RIRS residual stones often serve as infection foci, whereas the FANS reduces recurrence rates by attenuating intrapelvic pressure and minimizing biofilm formation on fragment surfaces (30, 31). In addition, the synergistic effect between the micronized fragmentation characteristics of the TFL laser and the FANS may enhance long-term clearance outcomes, although further data are required to validate this hypothesis (36, 37, 38). These findings underscore the multitemporal clearance efficacy of the FANS and its potential to redefine postoperative management paradigms in RIRS.

### Strengths and limitations

This article represents the first comparative analysis of the FANS versus the T-UAS in the treatment of urinary calculi. By incorporating subgroup analyses based on multiple variables including stone size and laser type, this study effectively integrated bias risk assessments and ensured the robustness of its findings. However, several limitations warrant attention. First, significant heterogeneity existed among the included studies, likely attributed to differences in study design, geographical regions, equipment used, and surgeon experience. Second, although non-significant, the limited number of studies evaluating certain outcome measures—such as hospital stay—may have influenced the accuracy of conclusions. These findings highlight the need for standardized protocols and multicenter trials to further validate the clinical efficacy of the FANS across diverse populations and practice settings.

## Conclusions

Findings from this study indicate that the FANS significantly enhances the efficacy and safety of RIRS by improving stone clearance rates, reducing complications, and minimizing reliance on adjunctive devices. Notably, its advantages in the management of larger calculi and laser compatibility represent critical clinical advancements. These results suggest that the FANS may potentially supplant PCNL for select patients, offering greater clinical benefits to patients, while aligning with the trend toward minimally invasive, natural orifice approaches.

## Data Availability

The original contributions presented in the study are included in the article/Supplementary Material, further inquiries can be directed to the corresponding author.
